# 

**Published:** 1899-12-02

**Authors:** 


					THE HOSPITAL. "The standard of highest purity."?Lancet. December 2xn, 1899.
CADBURY'S f Hf CADBURY'S
^ cogoa % MM JggLp cocoa
abmmt TTmn*? ^ Nf) HPTTnt HD AT VAT TT?Q TTOUH
ABSOLUTELY PURE.   ^ NO ?RUO? OR ALKALIES USED.
No. 688. Vol. XXVII. [JUgg-] Saturday,
Dec. 2nd, 1899.
seep?ri40?rms,J PRICE TWOPENCE.

				

